# The IL-33 Receptor/ST2 acts as a positive regulator of functional mouse bone marrow hematopoietic stem and progenitor cells

**DOI:** 10.1016/j.bcmd.2020.102435

**Published:** 2020-09

**Authors:** Maegan L. Capitano, Brad Griesenauer, Bin Guo, Scott Cooper, Sophie Paczesny, Hal E. Broxmeyer

**Affiliations:** a Department of Microbiology and Immunology, Indiana University School of Medicine, Indianapolis, IN, United States of America; b Department of Pediatrics, Indiana University School of Medicine, Indianapolis, IN, United States of America

**Keywords:** BM, bone marrow, HSC, hematopoietic stem cell, HPC, hematopoietic progenitor cell, WT, wildtype, LT, long-term, MPP, multipotent progenitor, CMP, common myeloid progenitor, GMP, granulocyte macrophage progenitor, MEP, megakaryocyte erythrocyte progenitor, rhEPO, recombinant human erythropoietin, rmSCF, recombinant mouse stem cell factor, CFU-GM, colony-forming unit- granulocyte macrophage, BFU-E, burst-forming unit- erythroid, CFU-GEMM, colony-forming unit- granulocyte erythrocyte macrophage megakaryocyte, [3H]Tdr, high specific activity tritiated thymidine, PB, peripheral blood, SDF-1, stromal derived factor 1, ST2, Hematopoietic stem cells, Hematopoietic progenitor cells, Hematopoiesis, Cytokine, Cytokine receptor

## Abstract

There is a paucity of information on a potential role for the IL-33 receptor/ST2 in the regulation of mouse bone marrow (BM) hematopoietic stem (HSC) and progenitor (HPC) cells. Comparing the BM of *st2*^−/−^ and wild type (WT) control mice using functional assays, it was found that *st2*^−/−^ BM cells had poorer engrafting capacity than WT BM in a competitive repopulating assay using congenic mice, with no changes in reconstitution of B-, T- and myeloid cells following transplantation. The BM of *st2*^−/−^ mice also had fewer granulocyte-macrophage, erythroid, and multipotential progenitors than that of WT BM and these *st2*^−/−^ HPC were in a slow cycling state compared to that of the rapidly cycling HPC of the WT mice. While functional assessment of HSC and HPC demonstrated that ST2 has a positive influence on regulation of HSC, we could not pick up differences in *st2*^−/−^ compared to WT BM using only phenotypic analysis of HSC and HPC populations prior to transplantation, again demonstrating that phenotypic analysis of HSC and HPC do not always recapitulate the functional assessments of these immature hematopoietic cells.

## Highlights

• ST2 is a positive modulator of hematopoiesis.
• *ST2^-/-^* is a positive modulator of hematopoiesis
• Immunophenotyping of *st2^-/-^* hematopoietic stem cell numbers does not recapitulate functional capability of these cells.

## 1 Introduction

There are numerous cytokines/growth factors/chemokines and their receptors that have been implicated in regulating hematopoietic stem (HSC) and progenitor (HPC) cells and hematopoiesis *in vivo* and *in vitro* [1]. There are likely more such ligand-receptor interactions that will be identified and found to be involved in such regulatory interactions. One is that of the IL-33/IL-33 Receptor (IL-33R; also known as ST2) axis. ST2 has been implicated in immune cell functions [2] including: autoimmune disorders [3], allergic inflammation [4], and has been used as a biomarker in acute graft vs. host disease [[5], [6], [7]]. It has been implicated in the pathogenesis of myeloproliferative disorders and hematologic malignancies and tumorgenesis [8,9]. However, there is a paucity of information on a role for ST2 in regulation of normal HSC/HPC and hematopoiesis, other than as a mobilizer of HSC/HPC to the blood of mice [10]. Herein, we evaluated the activities of HSC/HPC in *st2*^−/−^ mice using both phenotypic and functional assessments of HSC and HPC. We found that ST2 is involved in the positive regulation of hematopoiesis.

## 2 Methods and materials

### 2.1 Animals

C57BL/6J, Boy/J, B6 × Boy/J F1 mice (8–10 weeks old) were obtained from an on-site breeding core facility at Indiana University School of Medicine. *st2*^−/−^ mice (C57BL/6 background) were provided by Dr. Andrew McKenzie from University of Cambridge, UK [2]. *st2*^−/−^ mice were bred in the mouse breeding facility at Indiana University School of Medicine. Animals were maintained under temperature- and light-controlled conditions (21–24 °C, 12-h light/12-h dark cycle) and were group-housed according to age, sex, and genotype. Mice were fed ad libitum. For all experiments, mice were matched by age and sex. All animal protocols were approved by Institutional Animal Care and Use Committee at Indiana University School of Medicine.

### 2.2 Phenotypic analysis of HSC/HPC

For analyzing HSC and HPC phenotypes, BM cells were collected at a concentration of ~2.5 × 10^6^ cells per tube, washed in PBS, incubated in fluorescently-conjugated antibody cocktail for 15 min at room temperature, washed in PBS, and then fixed in 1.5% formaldehyde. The samples were analyzed on an LSRII flow cytometer (BD Biosciences). Single color compensation and isotype controls were included for each experiment. Data analysis was performed using FlowJo 7.6.3 software (TreeStar, WA, USA). Gates were determined using fluorescence minus-one controls. The percent of each population was used to calculate the absolute number of each population per femur. The following mouse phenotyping markers were used: FITC-mouse lineage cocktail (CD3, Gr-1, CD11b, CD45R, Ter119; BioLegend; cat. # 133302), PE-CF594-anti-Ly6A/E (a.k.a. Sca-1; clone D7; BD Biosciences), APC-H7-anti-CD117 (a.k.a. c-Kit; clone 2B8; BD Biosciences), BV421 or APC-anti-CD135 (a.k.a. Flt3; clone A2F10.1; BD Biosciences), BV421 or PE-anti-CD34 (clone RAM34; BD Biosciences), and PerCP-Cy™5.5-anti-CD16/CD32 (FcγR; clone 2.4G2; BD Biosciences). HSC and HPC populations for mice were defined as follows: LSK cells: Lin^−^ Sca-1^+^ c-Kit^+^, HSC: LSK CD34^−^, long-term (LT)-HSC: LSK Flt3^−^ CD34^−^, multipotent progenitor (MPP): LSK Flt3^+^ CD34^+^, LK cells: Lin^−^ Sca-1^−^ c-Kit^+^, common myeloid progenitor (CMP): LK CD34^+^ CD16/CD32^int^, granulocyte macrophage progenitor (GMP): LK CD34^+^ CD16/CD32^hi^, and megakaryocyte erythrocyte progenitor (MEP): LK CD34^−/lo^ CD16/CD32^−/lo^. For analysis of transplantation experiments, APC-anti-CD45.2 (clone 104), PE- or FITC-anti-CD45.1 (clone A20), PerCPCy™5.5-anti-CD45R/B220 (clone RA3-6B2), BV421-anti-CD3ε (clone 145-2C11), and PE-Cy™7-anti-CD11b (clone M1/70) were purchased from BD Biosciences. For all antibodies used in these studies, the validation for the relevant species and applications can be found on the indicated manufacturer's website.

### 2.3 Analysis of mRNA Levels of *actin*, *st2* and *cxcr4* in C57BL/6 Mice

C57BL/6 BM HSC, GMP and MEP cells were sorted using a FACSAria instrument (BD Biosciences). Total RNA was isolated from sorted cells using the RNeasy Plus Mini Kit (QIAGEN). cDNA was prepared with a SuperScript VILO cDNA Synthesis Kit (Invitrogen). qPCR was performed using SYBR Green PCR mix on an ABI Prism 7500HT (Applied Biosystems). Thermocycler conditions included 2-min incubation at 50 °C, then 95 °C for 10 min; this was followed by a 2-step PCR program of 95 °C for 5 s and 60 °C for 60 s for 40 cycles. β-Actin was used as an internal control. The primer sequences were as follows: *actin* forward 5′-CTCTGGCTCCTAGCACCATGAAGA-3′, reverse 5′-GTAAAACGCAGCTCAGTAACAGTCCG-3′; *st2* forward 5′-AAGGCACACCATAAGGCTGA-3′, reverse 5′-TCGTAGAGCTTGCCATCGTT-3′; *cxcr4* forward 5’-TCCTCCTGACTATACCTGACTTCATCT-3′, reverse 5’-CCTGTCATCCCCCTGACTGAT-3′.

### 2.4 Functional analysis of HPC

BM cells flushed from the femurs of the indicated mouse strain were plated at 5 × 10^4^ cells/mL in 1% methylcellulose culture medium with 0.1 mM hemin (Sigma-Aldrich; St. Louis, MO, USA), 30% FBS, 1 U/mL recombinant human erythropoietin (rhEPO; Amgen; Thousand Oaks, CA, USA), 50 ng/mL recombinant mouse stem cell factor (rmSCF; R&D Systems; cat. # 455-MC), and 5% vol/vol pokeweed mitogen mouse spleen cell conditioned medium. Colonies were scored after 6 days of incubation at 5% CO_2_ and lowered 5% O_2_ in a humidified chamber, and granulocyte-macrophage colony-forming units (CFU-GM), erythrocyte burst-forming units (BFU-E), and granulocyte, erythrocyte, macrophage, megakaryocyte colony-forming units (CFU-GEMM) progenitors were distinguished by examining the morphology of colonies. Total number of colonies per femur were calculated.

### 2.5 High specific activity triated thymidine kill assay

High specific activity tritiated thymidine ([3H]Tdr) kill assays were utilized to determine the percent HPC in cycle [13]. BM cells were treated with 50 μCi of high specific activity [3H]Tdr (20 Ci/mmol; DuPont NEN) at room temperature for 40 min then washed twice immediately prior to use in HPC colony assays. The number of colonies in the [3H]Tdr treated plates was then compared to vehicle control treated plates and the percent colonies in S-phase was calculated.

### 2.6 Functional analysis of HSC

Competitive transplantations were performed using donor BM cells from WT or *st2*^−/−^ mice. These cells were mixed in a 1:1 ratio (1 × 10^5^ cells) with competitor BM from Boy/J mice and injected intravenously into B6 × Boy/J F1 host mice that had been lethally irradiated (950 cGy) 24 h prior to transplantation. Following 1.5 and 5 months, the percentage of donor cells in the peripheral blood (PB) and BM was determined by flow cytometry. At each time point, PB and BM was collected from B6 x Boy/J F1 host mice and analyzed for percent donor cell engraftment of B cells (B220^+^), T cells (CD3ε^+^), and myeloid cells (CD11b^+^). In the BM (at each time point), donor LT-HSC, MPP, CMP, GMP and MEP numbers were determined by flow cytometry.

### 2.7 Statistics

Results are expressed as average mean values ± standard error mean (SEM) as indicated. Statistical analysis was performed using Microsoft Excel and GraphPad Prism 5.0. Two-tailed Student's *t-*test was used where indicated. A *P* value < 0.05 was considered statistically significant.

## 3 Results

### 3.1 C57BL/6 BM HSC and HPC Express *st2*

**Fig. 1:**
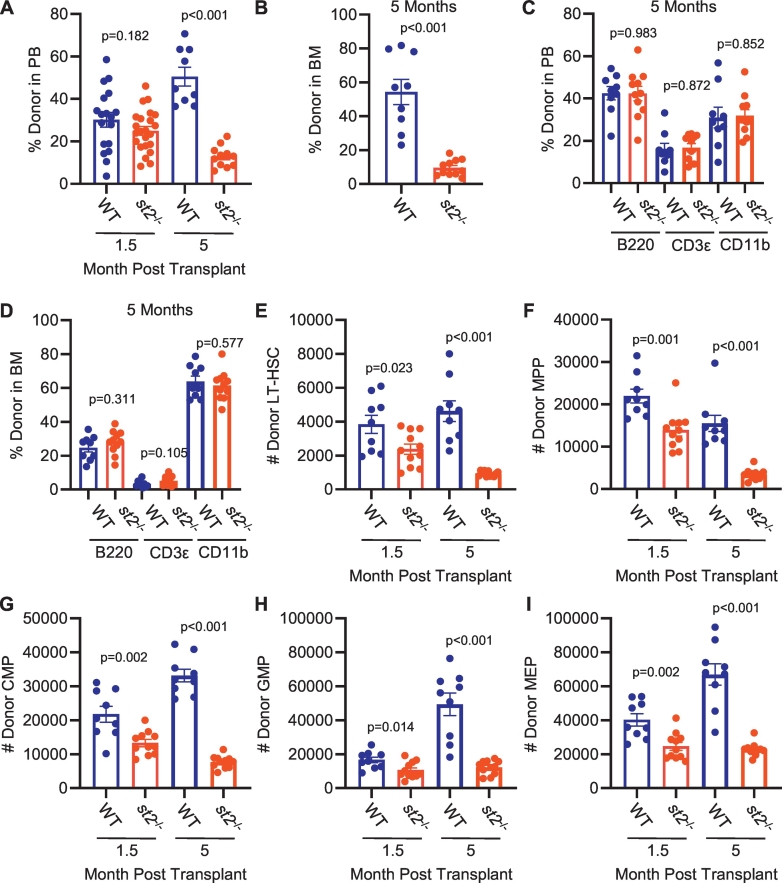
Bone marrow (BM) from *st2*^−/−^ mice demonstrates engrafting deficiencies in competitive BM transplantation analysis. Donor wildtype and *st2*^−/−^ BM was mixed in a 1:1 ratio with competitor BM cells and then injected i.v. into lethally irradiated B6 × Boy/J F1 mice. At 1.5- and 5-months post transplantation, percent donor cells in peripheral blood (PB) was determined by flow cytometry (A). At 5-months, the percent donor cells in the BM (B) and the number of donor B cells (B220^+^), T cells (CD3ε^+^), and myeloid cells (CD11b^+^) in the PB (C) and in the BM (D) was determined by flow cytometry. In the BM (at each time point after transplantation), donor long-term hematopoietic stem cell (LT-HSC; E), multipotent progenitor (MPP; F), common myeloid progenitor (CMP; G), granulocyte monocyte progenitor (GMP; H) and megakaryocyte erythrocyte progenitor (MEP; I) numbers were determined by flow cytometry. (A-I) All data are the mean ± SEM (*n* = 9–22 recipient mice). Statistical analysis was performed using Student's *t-*test with all *p* < 0.05 considered statistically significant.

In order to determine if ST2 could be detected in immature hematopoietic cells, *st2* mRNA expression was examined by qPCR in purified phenotypic populations of mouse BM HSC, MEP, and GMP (S. Fig. 1). All population of HSC/HPC examined expressed *st2*. For a positive control, populations of HSC, MEP, and GMP were examined for *cxcr4* mRNA expression (S. Fig. 1). The receptor for stromal derived factor-1 (SDF-1), CXCR4, has been implicated in chemotaxis [11] and homing [12] of HSC and HPC to the BM.

### 3.2 Phenotypic and functional analysis of WT C57BL/6 and *st2*^−/−^ mouse BM

**Fig. 2:**
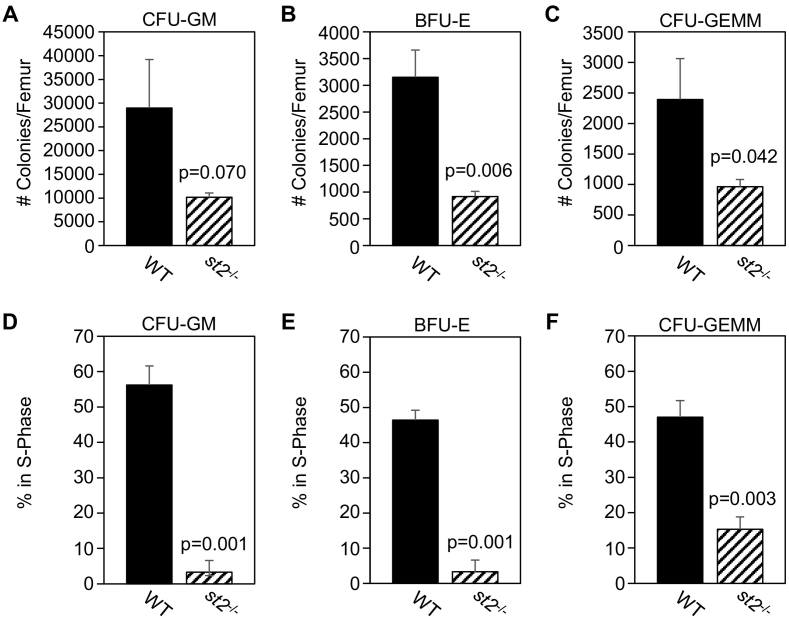
BM from *st2*^−/−^ mice have fewer functional hematopoietic progenitor cells (HPC). BM cells from wildtype and *st2*^−/−^ mice was utilized for HPC colony forming assays. (A-C) The number of colony-forming units- granulocyte, macrophage (CFU-GM; A) burst-forming unit-erythroid (BFU-E; B) and CFU- granulocyte, erythrocyte, macrophage, megakaryocyte (CFU-GEMM; C) per femur was determined. (D-F) Percentage of CFU-GM (D), BFU-E (E), and CFU-GEMM (F) in the S phase of the cell cycle was determined using the high-specific-activity tritiated thymidine kill technique. (A-F) Data are average mean ± SEM of 3 mice plated in triplicate. Statistical analysis was performed using Student's t-test.

To determine if the ST2/IL-33 axis regulates hematopoiesis under steady state conditions, HSC and HPC numbers were examined in the BM of *st2*^−/−^ versus wildtype mice. Numbers of phenotypically-defined LT-HSC, MPP, CMP, and GMP per femur were not statistically different between WT and *st2*^−/−^ mice (S. Fig. 2). However, it has been well established that phenotypical analysis of HSC and HPC numbers do not always recapitulate the number of HSC and HPC when assessed functionally [14]. Hence, we preceded to assess the functional capacities of HSC by engrafting studies in lethally-irradiated mice using competitive repopulating assays in congenic mice with WT C57BL/6 and *st2*^−/−^ donor BM cells, Boy/J competitor cells, and B6 x Boy/J F1 recipients. While there were no differences in PB donor cell chimerism between the WT and *st2*^−/−^ mice short-term at month 1.5 after engraftment (Fig. 1A) there was a large decrease in *st2*^−/−^ engraftment compared to WT cells in PB (Fig. 1A) and BM (Fig. 1B) at 5 months post-transplant, with no difference in donor-derived B, T, and myeloid cells in the PB (Fig. 1C) and BM (Fig. 1D). When we assessed the BM compartment of recipient mice at the 1.5 month timepoint following transplantation, we did see significant decreases in absolute numbers of *st2*^−/−^ donor-derived LT-HSC (1.62-fold decrease), MPP (1.58-fold decrease), CMP (1.64-fold decrease), GMP (1.57-fold decrease), and MEP (1.63 fold-decrease) compared to WT donor BM (Fig. 1E-I) which suggested to us that engraftment deficiencies may be seen at later timepoints. At 5 months post-transplant, the *st2*^−/−^ BM had even greater decreases in absolute numbers of *st2*^−/−^ donor-derived LT-HSC (4.96-fold decrease), MPP (4.53-fold decrease), CMP (4.29-fold decrease), GMP (4.04-fold decrease), and MEP (2.91-fold decrease). Thus, *st2*^−/−^ donor BM cells had greatly decreased numbers of functionally engrafting HSC compared to WT control donor BM cells.

### 3.3 Functional assessment of WT C57BL/6 and *st2*^−/−^ mouse BM HPC by colony assays

Functional numbers and cycling status of CFU-GM, BFU-E, and CFU-GEMM were assessed by colony assays. On evaluating numbers of CFU-GM, BFU-E, and CFU-GEMM, the *st2*^−/−^ BM had significantly decreased absolute numbers per femur of CFU-GEMM and BFU-E with a trend to decreased numbers of CFU-GM (Fig. 2A-C). Moreover, the CFU-GM, BFU-E, and CFU-GEMM from *st2*^−/−^ BM had significantly fewer progenitors in cycle than did WT cells (Fig. 2D-F). Thus, HPC numbers and cycling from *st2*^−/−^ BM were greatly reduced compared to that of control WT cells.

## 4 Discussion/conclusions

While there is increasing knowledge of functional consequences of the IL-33/ST2 axis for immunological events and for ST2 as a biomolecular marker [[2], [3], [4], [5], [6], [7], [8]], there is a paucity of information on a potential role for ST2 in the regulation of hematopoiesis, with one publication stating that IL-33, the ligand for ST2, can act as a mobilizer of HSC and HPC [10]. However, this paper only evaluated mobilized HSC based on phenotypic surface markers, and it is known that phenotype does not always recapitulate the functional capabilities of these cells, especially under conditions of stress [14]. We have now shown that while phenotypic analysis of HSC and HPC did not detect differences between numbers of these cells in the BM of *st2*^−/−^ and WT cells prior to transplantation, upon functional analysis of these cells there was significantly decreased engraftment of *st2*^−/−^ BM and fewer functional *st2*^−/−^ BM CFU-GM, BFU-E, and CFU-GEMM per femur compared to WT BM. Moreover, the CFU-GM, BFU-E, and CFU-GEMM of *st2*^−/−^ BM were in a relatively slow cycling status compared to the increased cycling status of the functionally assessed HPC of the control WT cells. Thus, *st2* acts as a positive modulator of functional HSC and HPC. Future studies in this area are likely to shed more light on a role of the IL-33/ST2 (IL-33R) axis on regulating hematopoiesis.

## Appendix A Supplementary data

Supplementary figures Image 1
