# Supplementary material for: The IL-33 Receptor/ST2 acts as a positive regulator of functional mouse bone marrow hematopoietic stem and progenitor cells
Source: Blood Cells Mol Dis. 2020 Sep;84:102435. doi: 10.1016/j.bcmd.2020.102435 (PMC7788514; doi:10.1016/j.bcmd.2020.102435)

Supplemental Material

Supplemental Figure 1. C57BL/6 bone marrow (BM) hematopoietic stem (HSC) and progenitor (HPC) cells express *st2*. HSC (Lin$^-$ Sca-1$^+$ c-Kit$^+$ [LSK] CD34$^-$), granulocyte macrophage progenitor (GMP; Lin$^-$ Sca-1$^-$ c-Kit$^+$ [LK] CD34$^+$ CD16/CD32^{hi}), and megakaryocyte erythrocyte progenitor (MEP; LK CD34$^{-/lo}$ CD16/CD32$^{-/lo}$) populations were sorted and examined for *st2* expression by qPCR. For positive controls, *actin* and *cxcr4* (a chemokine receptor known to be expressed on HSC and HPC populations) were examined as well. Image is a representative of four replicates.

Supplemental Figure 2. BM from *st2*$^{-/-}$ mice show no phenotypic abnormalities in the HSC and HPC compartment under baseline conditions. (**A-F**) Immunophenotyping of BM harvested from wildtype (WT; n=3) and *st2*$^{-/-}$ (n=3) mice was performed to examine long-term (LT)-HSC (LSK Flt3$^-$ CD34$^-$; **B**), multipotent progenitor (MPP; LSK Flt3$^+$ CD34$^+$; **C**), common myeloid progenitor (CMP; LK CD34$^+$ CD16/CD32^{int}; **D**), GMP (**E**), and MEP (**F**) populations using flow cytometry. Data are mean ± SEM. Statistical analysis was performed using Student's t test with all $p<0.05$ considered statistically significant.

Capitano et al., Supplemental Figure 1

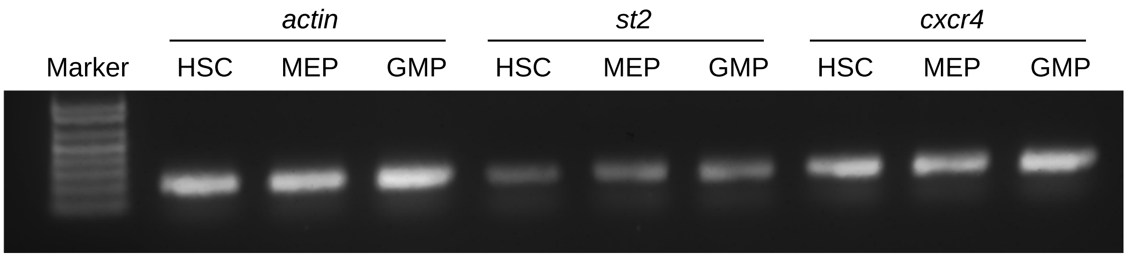

Capitano et al., Supplemental Figure 2

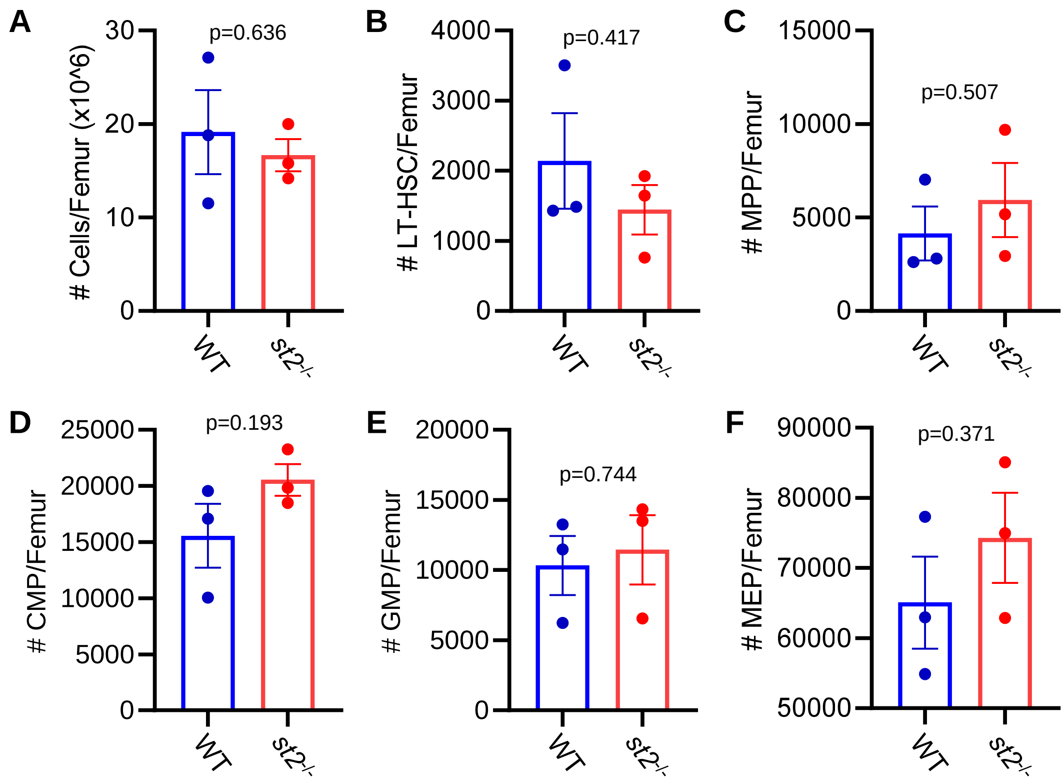

Supplement: Supplementary file 1 — Supplementary figures [file mmc1.pdf]
